# Optogenetic inhibition of neurons by internal light production

**DOI:** 10.3389/fnbeh.2014.00108

**Published:** 2014-04-01

**Authors:** Benjamin B. Land, Catherine E. Brayton, Kara E. Furman, Zoe LaPalombara, Ralph J. DiLeone

**Affiliations:** Department of Psychiatry, Yale University School of MedicineNew Haven, CT, USA

**Keywords:** optogenetics, dopamine, luciferase, amphetamine, striatum

## Abstract

Optogenetics is an extremely powerful tool for selective neuronal activation/inhibition and dissection of neural circuits. However, a limitation of *in vivo* optogenetics is that an animal must be tethered to an optical fiber for delivery of light. Here, we describe a new method for *in vivo*, optogenetic inhibition of neural activity using an internal, animal-generated light source based on firefly luciferase. Two adeno-associated viruses encoding luciferase were tested and both produced concentration-dependent light after administration of the substrate, luciferin. Mice were co-infected with halorhodopsin- and luciferase-expressing viruses in the striatum, and luciferin administration significantly reduced Fos activity compared to control animals infected with halorhodopsin only. Recordings of neuronal activity in behaving animals confirmed that firing was greatly reduced after luciferin administration. Finally, amphetamine-induced locomotor activity was reduced in halorhodopsin/luciferase mice pre-injected with luciferin compared to controls. This demonstrates that virally encoded luciferase is able to generate sufficient light to activate halorhodopsin and suppress neural activity and change behavior. This approach could be used to generate inhibition in response to activation of specific molecular pathways.

## Introduction

The use of optogenetics has increased dramatically in the last several years, largely due to its applicability to studies spanning from single-cell electrophysiology to whole-animal behavior. *In vivo* techniques are particularly powerful as they allow for control of behavior with manipulation of a single neuron subtype (Witten et al., 2010; Narayanan et al., 2012). However, one restriction to *in vivo* optogenetics is the necessity of an external light source (laser or LED) that must be connected to the animal via indwelling cannula. While optical rotary joints allow for relatively free movement within an open-topped behavioral apparatus, home-cage illumination or any structure with an enclosed top are not feasible. Recently, two groups have introduced wireless optogenetics approaches, where the signal/power is sent to a headstage containing indwelling LEDs (Wentz et al., 2011; Kim et al., 2013). While this solves the problem of tethering, specialized equipment is needed to both send the signal and to receive it, which could add a significant cost to the setup and may require significant expertise to implement.

One potential solution is to eliminate the need for an external light source by creating a system in which the animal produces its own light. This can be accomplished by leveraging light-producing proteins that exist in nature, in a similar fashion to the opsins that are sensitive to light. These proteins can be expressed using viral systems in a way similar to the opsins, to internally generate light. Firefly luciferase is one such protein, and it produces yellow-red light in the presence of its substrate, luciferin (Nakatsu et al., 2006). Mammalian systems readily tolerate luciferase and luciferin, and this system has been used *in vivo* to measure gene activity and track labeled cells (Contag, 2007). Notably, the emission spectrum of firefly luciferase overlaps highly with the action spectrum of halorhodopsin (Zhang et al., 2007), a bacterially derived, amber light photoreceptive chloride pump that inhibits neural activity. This suggests that with sufficient light output, luciferase could serve as a light source that would activate halorhodopsin and inhibit neural activity, eliminating the need for an external light source, and establishing a genetically-encoded light generating system. While this reduces control over timing, it would allow for production of light in response to specific cellular signals. Here, we show that luciferin/luciferase and halorhodopsin are sufficient to reduce neural activity *in vivo* and disrupt basic behaviors, establishing a proof-of-principle for luciferase-based inhibition.

## Materials and methods

### Animals

Eighteen male C57Bl/6 mice were used for these studies. Animals were group housed, with the exception of the two mice used for electrophysiology that were single-housed after surgery. Animals were kept on a 12 h light/dark cycle and provided standard chow and water *ad libitum*, and all animal procedures were performed in accordance with the protocol approved by the Institutional Animal Care and Use Committee (IACUC).

### Viral preparation and surgery

Viral production for the EF1a-luciferase construct was accomplished using a triple-transfection, helper-free method, and purified as described in detail previously (Hommel et al., 2003). To generate the EF1a-luciferase construct, a flox-ChR2 construct (AAV2-DIO-ChR2-eYFP, UNC viral core) was restriction digested outside of the asymmetric loxP sites using NheI and AscI restriction enzymes, and a cassette containing luciferase was inserted. AAV5-hSyn-eNpHR3.0-eYFP (halorhodopsin) and AAV2-CMV-luciferase virus were purchased from University of North Carolina viral core.

For surgery, animals were anesthetized with 10% ketamine/ 1% xylazine (10 ml/kg body weight) and placed in a stereotaxic frame (Stoelting). After craniotomy, mice were injected with AAV2-luciferase (0.5 uL, EF1a or CMV) and/or AAV2-halorhodopsin (0.5 uL) into the dorsal striatum (AP: +0.7, ML ± 1.75, DV −3.5 from bregma). For the electrophysiological studies, animals were injected with virus into the ventral striatum (AP: +1.4, ML −0.7, DV −4.5 from bregma) before placement of the electrode. A 16-channel array of microwire electrodes (Tucker-Davis Technologies, TDT) was placed in the same craniotomy. A ground wire attached to the array was placed above the dura through a burr hole in the opposite hemisphere. Arrays were composed of 16 Teflon-coated, 50 μm tungsten wires arranged in an 8 × 2 configuration with each electrode spaced by 250 μm. *In vitro* impedance of the electrodes is 100–300 kohms. Electrodes were affixed to the skull with cyanoacrylate (“Metabond”) and methyl methacrylate (dental cement) and two screws placed over the parietal bones. In total, headstages weighed approximately 2.5 g and were well-tolerated by animals.

### *In vitro* light assay

HT-1080 cells were cultured using standard media, and were infected with either no virus or increasing dilutions of the EF1a or CMV luciferase virus (1:100, 1:500, 1:2500). Twenty four hours after infection, cells were harvested and combined with a luciferase based light detection kit (Stratagene). After adding luciferin, the cells were placed in a luminometer (Berthold) and light was detected, expressed as Relative Light Units per second (RLU/s).

### *In vivo* electrophysiology

After approximately 2–3 weeks of recovery to ensure optimal viral expression and neuronal signal, freely moving animals were connected to the recording system while in a behavioral box (Med Associates). After 1 h of habituation, neurons were recorded for a 10 min baseline and then given an IP injection of either saline or luciferin (150 mg/kg). Recordings were continued for 1 h post-injection. Plugging and unplugging from the electrode cables was performed under isofluroane anesthesia to minimize stress on the animals.

Neural ensemble recordings were made using a multichannel acquisition processor (MAP) from TDT. Putative single neural units were identified on-line using an oscilloscope and an audio monitor. The TDT off-line sorter was used to analyze the signals and to remove artifacts due to cable noise. Principal component analysis (PCA) and waveform shape were used for spike sorting (Sears et al., 2010). Single units were identified as having: (1) consistent waveform shape; (2) separable clusters in PCA space; (3) average amplitude estimated at least three times larger than background activity; and (4) a consistent refractory period of at least 2 ms in interspike interval histograms. Those units identified on-line as potential single units that did not meet these criteria off-line were not included in this analysis. Graphical exploratory analysis of neural activity and quantitative analysis of basic firing properties (firing rate, interspike intervals) were analyzed using custom routines for MATLAB. For each well isolated neuron, post-injection firing rates were normalized to mean pre-injection firing rates (in the 10 min immediately preceding drug injection) and binned (60 s bins). Activity was then compared between neurons recorded in luciferin and saline conditions.

### Locomotor activity

After 2–3 weeks of recovery after viral surgery, animals were pre-habituated for 90 min to locomotor chambers that consisted of a mouse cage-bottom, without bedding, inside of an infrared one-dimension beam-break array (Med Associates). On test day, animals were again habituated to the chambers for 90 min. Animals were then briefly removed and injected with luciferin (150 mg/kg) and returned to the chamber. Twenty minutes later animals were injected with amphetamine (2.5 mg/kg) and returned to the chamber for 90 min. Locomotor counts were calculated as the number of consecutive beam-breaks in the chamber, and these counts were made throughout the entire testing period.

### Immunofluorescence

Animals that performed the locomotor behavioral tests were allowed to recover for at least 2 weeks before sacrifice. On the day of sacrifice, animals were injected with luciferin (150 mg/kg), followed 20 min later by injection of pentylenetetrazol (PTZ, 45 mg/kg) to induce seizure activity. Animals were observed for signs of seizure (as confirmation of a general increase in neural activity) and 90 min after PTZ injection were deeply anesthetized and intracardially perfused with 4% paraformaldehyde. The brain was removed and post-fixed overnight in paraformaldehyde, and after immersion in sucrose for cryoprotection, 40 μm sections were made on a freezing microtome, and stored in 1 × PBS with 0.01% sodium azide to prevent bacterial growth. Immunohistochemistry was performed according to methods described previously (Sears et al., 2010). Staining for Fos (Rabbit anti-cFos; Santa Cruz; 1:500) or Luciferase (Goat anti-luciferase; Promega; 1:500) with secondary antibodies (Alexa or 555 and 633; Invitrogen/Life Sciences) was performed in 3% normal donkey serum and 0.3% Triton-X 100. Tissue was visualized and images were captured using a fluorescent microscope (Zeiss) using standard FITC and TRITC filters or using a confocal microscope (Olympus). Fos labeling was quantified by standard threshold settings in ImageJ (NIH) over matched areas on one or more (averaged) sections per animal.

### Statistics

Comparisons were made using one-way and two-way ANOVA, and unpaired, two-tailed *t*-tests where appropriate. Differences in means were considered significant if *p*-values were <0.05, and were calculated using Graphpad Prism 5.0 and 6.0. Error bars represent s.e.m.

## Results

**Figure 1:**
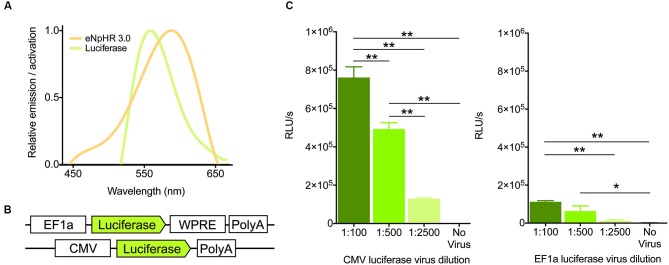
**Virally expressed luciferase generates light. (A)** Emission and activation spectra for firefly luciferase (green) and halorhodopsin (amber), respectively. Note the high degree of overlap between the two. **(B)** Schematics of the firefly luciferase viral constructs used. **(C)** Light output (in Relative Light Units per second) of the two viral constructs after 24 h of infection at various dilutions of virus in HT-1080 cells (*n* = 4 per group for CMV, *n* = 5,6,5,7 for 1:100, 1:500, 1:2500, no virus respectively for EF1a). * *P* < 0.05, ** *P* < 0.01.

Because the emission spectrum of firefly luciferase overlaps considerably with the action spectrum of halorhodopsin (Figure 1A), we reasoned that light generation from luciferase should activate these chloride pumps to drive hyperpolarization of membranes. Towards this goal, we designed an adeno-associated viral construct containing the firefly luciferase gene with an EF1a promoter, in addition to testing a virus containing the CMV promoter (UNC viral core, Figure 1B). To assess the ability of these constructs to generate light *in vitro*, we infected HT-1080 cells with varying dilutions. After 24 h of infection, cells were assayed for light production after treatment with luciferin. With both viruses, light production was dilution-dependent, with higher concentration of virus producing more light, as expected (Figure 1C, CMV, *F*_(3,12)_ = 113.7, *P* < 0.0001; EF1a, *F*_(3,19)_ = 12.01, *P* = 0.0001). The CMV-luciferase generated approximately 6-fold more light (0.78 order of magnitude) than the EF1a-luciferase, likely due to differences in titer between the viruses. Nonetheless, these results suggest that both constructs are capable of producing light.

**Figure 2:**
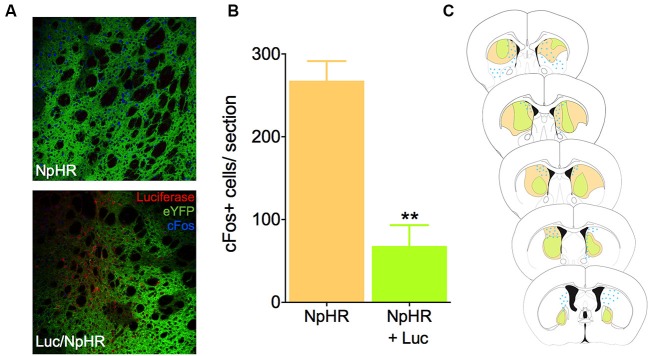
**Luciferase and halorhodopsin inhibit cFos activity. (A)** Micrographs depicting the dorsal striatum of an animal infected with luciferase (red) and halorhodopsin (green, lower), or halorhodopsin alone (upper). cFos immunofluorescence 90 min after PTZ injection is in blue. **(B)** Quantification of cFos in the dorsal striatum of luciferase/halorhodopsin and halo only animals 90 min after PTZ injection (*n* = 6 striatal samples from four animals). **(C)** Schematic drawing of the rostro-caudal distribution of luciferase, halorhodopsin, and cFos in one animal. Note that in general, there is very little cFos immunofluorescence where both luciferase and halorhodopsin are expressed. ** *P* < 0.01.

To test whether luciferase-induced light was sufficient to inhibit neural activity *in vivo* using biochemical markers, we infected mice in the dorsal striatum with either a combination of the EF1a-luciferase and halorhodopsin, or halorhodopsin alone. After recovery and behavioral testing (described below), animals were pre-treated with luciferin (150 mg/kg) 20 min prior to injection with PTZ, a drug known to induce seizures and cFos expression throughout the striatum. This dose of luciferin was derived from literature showing luciferase activity for 1–2 h after injection (Burgos et al., 2003; Cordeau and Kriz, 2012). Ninety minutes after PTZ injection, animals were sacrificed and processed for cFos immunofluorescence. Control animals injected with halorhodopsin alone showed a robust Fos induction in the dorsal striatum, and this response was reduced when luciferase was co-expressed with halorhodopsin (Figures 2A, B, *t*_10_ = 5.78, *P* = 0.0002). We mapped the extent of both viral expression and cFos induction (Figure 2C), and found a large volume of viral spread and a striking reduction in cFos in regions with expression of both viruses. These results suggest that luciferase is able to produce light *in vivo* in at sufficient power to activate halorhodopsin and affect neural activity.

**Figure 3:**
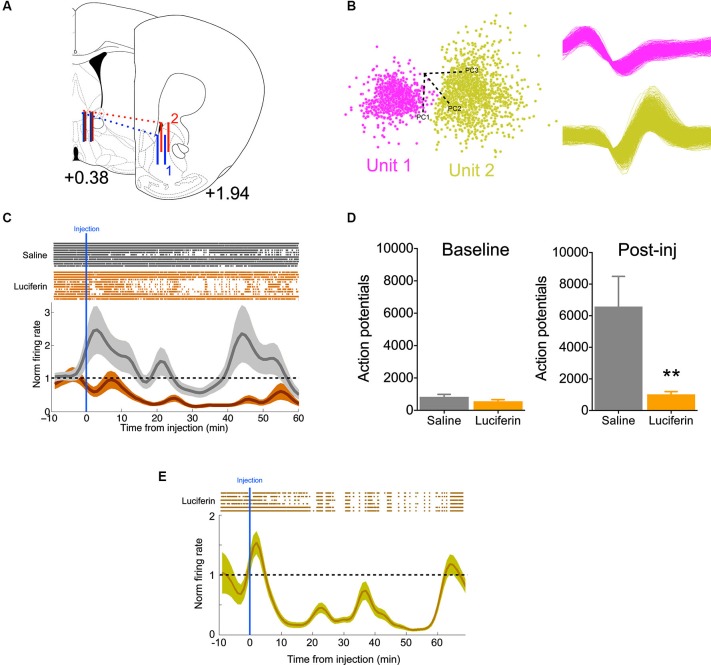
**Individual neurons show decreased activity during luciferase-based illumination. (A)** Schematic showing the rostral-caudal position of the 16-channel electrode for two animals (blue and red). Solid vertical lines represent the first and last pair of electrodes in the 2 × 8 array (modified from Paxinos and Franklin, 2004; values are AP distance from bregma in mm). **(B)** Left, two neurons sorted by principle component analysis (PCA) in one channel. Axes refer to the different principle components. Right, the two waveforms corresponding to the PCA. **(C)** Top, rastors depicting action potentials for neurons in the saline (gray, *n* = 11) and luciferin (orange, *n* = 12) injected conditions over the timecourse of the experiment. Bottom, normalized firing rate over the same period. Note that while the saline condition remains around the baseline, luciferin injection produces a prolonged decrease in firing. **(D)** Left, total number of action potentials during the baseline period (10 min). Right, total number of action potentials after injection (60 min, *n* = 11,12 for saline and luciferin, respectively). **(E)** Top, rastors depicting action potentials for the luciferin-injected condition (*n* = 6). Bottom, normalized firing rate over the course of the experiment. ** *P* < 0.01.

To further demonstrate the efficacy of this inhibitory strategy in real time, we implanted mice with 16-channel electrodes for *in vivo* electrophysiology, in addition to the luciferase/halorhodopsin dual infection, into the ventral striatum (Figure 3A). This allowed us to directly monitor neural activity as a function of luciferin/luciferase-driven light. Following acclimation to the chamber, animals were given a 10 min baseline period, after which they were injected with either luciferin (150 mg/kg), or saline on alternating, counterbalanced days. Units were sorted by PCA based on waveform (Figure 3B), and firing was normalized to the baseline period. Compared to saline injection, luciferin produced a marked decrease in firing that began soon after injection (Figure 3C, significantly different from normalized firing = 1 at 9 min, one-sample *t*-test, *P* < 0.01) and lasted at least 60 min (*F*_(69,1449)_ = 3.61, *P* < 0.0001, interaction of time by treatment). Summing total action potentials shows that during the baseline period (−10–0 min) there was no difference in spikes (*t*_21_ = 1.47, *P* = 0.16), while there was a dramatic decrease in spiking in the 60 min after luciferin injection compared to saline (Figure 3D; *t*_21_ = 3.03, *P* = 0.006). Because we did not see a return to baseline, a third animal was implanted as described above and injected with a 10-fold lower dose of luciferin (15 mg/kg). Again, we saw a characteristic decrease in firing, but at this dose the normalized firing returned to baseline at 60 min (Figure 3E). This shows that neural activity can be attenuated rapidly and in a sustained fashion using this luciferase-based approach.

**Figure 4:**
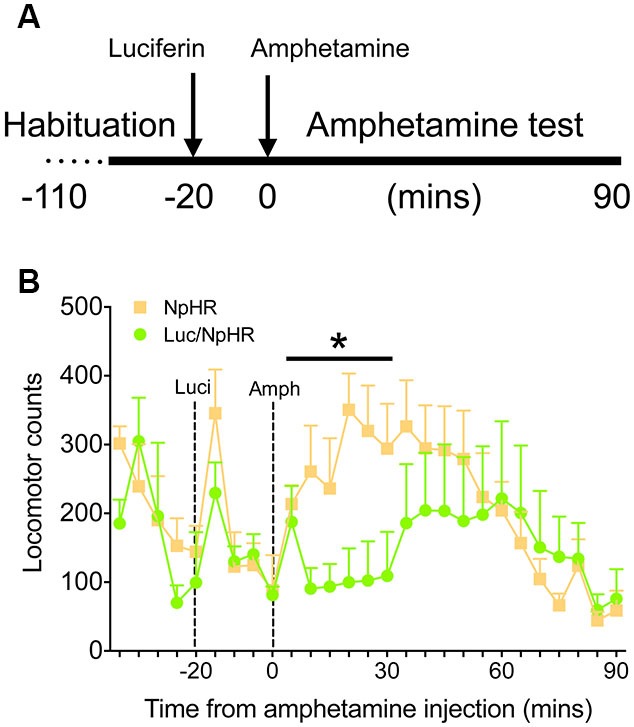
**Amphetamine induced locomotor activity is attenuated with luciferase-based illumination. (A)** Timeline of the behavioral test. **(B)** Locomotor activity during the last 20 min of baseline, 20 min of luciferin alone, and 90 min after amphetamine (*n* = 6 per group). * *P* < 0.05 for 30 min following amphetamine injection, two-way ANOVA, interaction of time and treatment.

Finally, to demonstrate that these decreases in activity have functional behavioral consequences, animals with bilateral, dorsal striatal injections of either luciferase/halorhodopsin or halorhodopsin alone were tested in a locomotor activity paradigm after amphetamine treatment. Animals were habituated to the locomotor boxes (90 min) on the testing day, and were then injected with luciferin (150 mg/kg) 20 min before injection of amphetamine (2.5 mg/kg, Figure 4A). While there were no differences in the last 20 min of the habituation period or the 20 min following luciferin injection, animals with luciferase/halorhodopsin expression were unaffected by amphetamine for 30 min after amphetamine injection, compared to controls whose activity increased as expected (Figure 4B; *F*_(5,50)_ = 2.83, *P* = 0.025). After 30 min, the experimental animals’ activity increased slightly and matched control activity for the remainder of the test period. These findings corroborate the neural activity data, and show that neural inhibition using this approach can have profound effects on behavior.

## Discussion

In the present study, we have demonstrated that a combination of luciferase and halorhodopsin viruses can be used together to suppress neural activity without an external light source. This hybrid, chemical-optogenetics approach requires administration of luciferin, but is otherwise noninvasive and could be easily adapted to any testing apparatus or environment. Potentially, luciferin could be delivered via minipump for chronic exposure. Our initial studies have shown that a single injection of luciferin can inhibit neurons for at least 1 h *in vivo*, and this correlates strongly with our behavioral measures. Further, this approach is adaptable to cell-type specific targeting using Cre-lox systems with halorhodopsin.

In principle, this technique resembles the DREADD (Designer Receptors Exclusively Activated by Designer Drugs) system for inactivating neurons (Armbruster et al., 2007), but there are at least two important differences. First, because this uses a combination of chemogenetics and optogenetics, one could also use traditional optogenetic illumination in these animals if needed. This makes the present system more adaptable to current optogenetic approaches and allows a single set of animals to be used with both external and internal light delivery. Also, because the halorhodopsin is a chloride pump, its inhibitory effects are rapid and direct with behavioral effects lasting <1 h. This is in contrast to the DREADD system, which uses G-protein coupled receptors whose behavioral effects last ~8 h after agonist (clozapine N-oxide) injection (Alexander et al., 2009; Carter et al., 2013). While the onset time for DREADD stimulation can also be rapid (e.g., Sasaki et al., 2011; Garner et al., 2012), the present data suggests that the luciferase system may provide a shorter time window of effect.

The use of a genetically encoded luciferase also allows potential application of this system as part of an inhibitory feedback loop in response to activation of a specific signaling pathway. Luciferase could be put under direct transcriptional regulation of a known pathway, or potentially modified to allow for post-translational activation, allowing for dynamic response that reflects activation of a signaling cascade. Although this approach would still require injection of luciferin, it could be useful for functional identification of pathways associated with addiction, or other diseases where aberrant neuronal activation contributes to disease pathology.

In sum, this endogenous light-production technique adds to the expanding toolkit that can be applied to optogenetic control of behavior. Uniquely, this approach allows neuronal inhibition without tethering or specialized equipment, expanding the breadth of behavioral experiments that can be performed.

## Conflict of interest statement

The authors declare that the research was conducted in the absence of any commercial or financial relationships that could be construed as a potential conflict of interest.

## References

[B1] Alexander G. M. Rogan S. C. Abbas A. I. Armbruster B. N. Pei Y. Allen J. A. (2009). Remote control of neuronal activity in transgenic mice expressing evolved G protein-coupled receptors. Neuron 63, 27–39 10.1016/j.neuron.2009.06.014 19607790 PMC2751885

[B2] Armbruster B. N. Li X. Pausch M. H. Herlitze S. Roth B. L. (2007). Evolving the lock to fit the key to create a family of G protein-coupled receptors potently activated by an inert ligand. Proc. Natl. Acad. Sci. U S A 104, 5163–5168 10.1073/pnas.0700293104 17360345 PMC1829280

[B3] Burgos J. S. Rosol M. Moats R. A. Khankaldyyan V. Kohn D. B. Nelson M. D. (2003). Time course of bioluminescent signal in orthotopic and heterotopic brain tumors in nude mice. Biotechniques 34, 1184–1188 12813886 10.2144/03346st01

[B4] Carter M. E. Soden M. E. Zweifel L. S. Palmiter R. D. (2013). Genetic identification of a neural circuit that suppresses appetite. Nature 503, 111–114 10.1038/nature12596 24121436 PMC3878302

[B5] Contag C. H. (2007). In vivo pathology: seeing with molecular specificity and cellular resolution in the living body. Annu. Rev. Pathol. 2, 277–305 10.1146/annurev.pathol.2.010506.091930 18039101

[B6] Cordeau P. Kriz J. (2012). Real-time imaging after cerebral ischemia: model systems for visualization of inflammation and neuronal repair. Methods Enzymol. 506, 117–133 10.1016/B978-0-12-391856-7.00031-7 22341222

[B16] Garner A. R. Rowland D. C. Hwang S. Y. Baumgaertel K. Roth B. L. Kentros C. (2012). Generation of a synthetic memory trace. Science 335, 1513–1516 10.1126/science.1214985 22442487 PMC3956300

[B7] Hommel J. D. Sears R. M. Georgescu D. Simmons D. L. Dileone R. J. (2003). Local gene knockdown in the brain using viral-mediated RNA interference. Nat. Med. 9, 1539–1544 10.1038/nm964 14634645

[B8] Kim T. I. McCall J. G. Jung Y. H. Huang X. Siuda E. R. Li Y. (2013). Injectable, cellular-scale optoelectronics with applications for wireless optogenetics. Science 340, 211–216 10.1126/science.1232437 23580530 PMC3769938

[B9] Nakatsu T. Ichiyama S. Hiratake J. Saldanha A. Kobashi N. Sakata K. (2006). Structural basis for the spectral difference in luciferase bioluminescence. Nature 440, 372–376 10.1038/nature04542 16541080

[B10] Narayanan N. S. Land B. B. Solder J. E. Deisseroth K. Dileone R. J. (2012). Prefrontal D1 dopamine signaling is required for temporal control. Proc. Natl. Acad. Sci. U S A 109, 20726–20731 10.1073/pnas.1211258109 23185016 PMC3528521

[B17] Paxinos G. Franklin K. B. J. (2004). The Mouse Brain in Stereotaxic Coordinates. Elsevier

[B15] Sasaki K. Suzuki M. Mieda M. Tsujino N. Roth B. Sakurai T. (2011). Pharmacogenetic modulation of orexin neurons alters sleep/wakefulness states in mice. PLoS One 6:e20360 10.1371/journal.pone.0020360 21647372 PMC3103553

[B11] Sears R. M. Liu R.-J. Narayanan N. S. Sharf R. Yeckel M. F. Laubach M. (2010). Regulation of nucleus accumbens activity by the hypothalamic neuropeptide melanin-concentrating hormone. J. Neurosci. 30, 8263–8273 10.1523/JNEUROSCI.5858-09.2010 20554878 PMC2907886

[B12] Wentz C. T. Bernstein J. G. Monahan P. Guerra A. Rodriguez A. Boyden E. S. (2011). A wirelessly powered and controlled device for optical neural control of freely-behaving animals. J. Neural Eng. 8:046021 10.1088/1741-2560/8/4/046021 21701058 PMC3151576

[B13] Witten I. B. Lin S.-C. Brodsky M. Prakash R. Diester I. Anikeeva P. (2010). Cholinergic interneurons control local circuit activity and cocaine conditioning. Science 330, 1677–1681 10.1126/science.1193771 21164015 PMC3142356

[B14] Zhang F. Wang L.-P. Brauner M. Liewald J. F. Kay K. Watzke N. (2007). Multimodal fast optical interrogation of neural circuitry. Nature 446, 633–639 10.1038/nature05744 17410168

